# 633 Marijuana Use in a Nationwide Survey of Burn Survivors

**DOI:** 10.1093/jbcr/iraf019.262

**Published:** 2025-04-01

**Authors:** Ana Reyes, Walter Ramsey, Christopher O’Neil, Michael Cobler-Lichter, Mary Ishii, Shevonne Satahoo, Joyce Kaufman, Louis Pizano, Tulay Koru-Sengul, Jose Szapocznik, Carl Schulman

**Affiliations:** University of Miami; University of Miami; University of Miami; University of Miami, Jackson Memorial Hospital; University of Miami Miller School of Medicine; University of Miami; University of Miami; University of Miami / Jackson Health System; University of Miami Miller School of Medicine; University of Miami; University of Miami

## Abstract

**Introduction:**

There is growing interest in the use of marijuana and its derivatives for treating a variety of symptoms including chronic neuropathic pain, sleep disturbances, and anxiety. However, patterns of marijuana use among burn survivors is unknown. The aim of this study was to examine marijuana use in a nationwide sample of burn survivors. We hypothesized that marijuana use would be frequent and common among survivors reporting pain.

**Methods:**

A survey was distributed through the Phoenix Society for Burn Survivors to adult burn survivors across the US (March-June 2023). The survey elicited demographics, burn history, the impact of various symptoms on quality of life, and treatments used to alleviate symptoms. Marijuana was listed as a treatment along with topical treatments, medications, mental health support, physical therapy, mindfulness techniques, and alternative medicine therapies. Chi-square test for association and multiple binary logistic regression analyses for marijuana use were performed. Adjusted odds-ratio (aOR) and 95% confidence intervals (95%CI) were calculated.

**Results:**

There were 178 survey respondents. The majority were female (60%), >54 years old (51%), White (75%), and had >20% total body surface area (TBSA) burns (75%). Twelve percent of survivors were < 1 year removed from injury, 17% 1-3 years, 11% 4-6 years, 7% 7-10 years, 14% 11-20 years, and 39% were >20 years removed. Marijuana use was reported by 41 (23%) of survivors. Significantly higher proportion of marijuana use was observed for survivors with visible burns compared with non-visible burns (29.9% vs. 10.3%, p=0.004). Survivors with burns to the forearms (28.7% vs. 14.3%, p=0.026) or hands (27.6% vs. 14.5%, p=0.048) had significantly higher proportion of marijuana use than survivors without injuries to those regions. There were no differences in marijuana use between survivors with and without injuries to the face, torso, upper arms, buttock, thighs, lower legs, feet, or genitals. On multiple regression, ‘debilitating or very negative’ impact of pain (aOR=11.61, 95%CI: 2.33-57.79, p=0.003) and ‘debilitating or very negative’ impact of poor body image (aOR=9.95, 95%CI: 1.03-96.29, p=0.047) on quality of life were associated with marijuana use. Age, TBSA, and the impact of sensation loss and heat/cold intolerance on quality of life were not associated with marijuana use.

**Conclusions:**

In this nationwide survey study, marijuana use was more common among burn survivors with visible burns and burns to the forearms or hands. Pain and poor body image were also associated with marijuana use. Future studies of efficacy and other effects of marijuana use in this population require further study.

**Applicability of Research to Practice:**

These findings provide insight into injured body regions and symptoms related to marijuana use in burn survivors.

**Funding for the Study:**

N/A

